# Neurotoxoplasmosis in the Immunocompetent: A Rare Occurrence

**DOI:** 10.7759/cureus.36782

**Published:** 2023-03-28

**Authors:** Saad Khalid, Shehzeen F Memon, Laraib Jumani, Shahzeb A Memon, Mishal S Siddiqui

**Affiliations:** 1 Department of Medicine, Dow University of Health Sciences, Civil Hospital Karachi, Karachi, PAK; 2 Department of Neurology, Dow University of Health Sciences, Civil Hospital Karachi, Karachi, PAK; 3 Department of Neurology, Pakistan Institute of Medical Sciences, Islamabad, PAK; 4 Department of Surgery, Dow University of Health Sciences, Civil Hospital Karachi, Karachi, PAK

**Keywords:** co-trimoxazole, aids, anti-toxoplasma antibodies, immunocompetent, toxoplasmosis

## Abstract

Cerebral toxoplasmosis is a rare condition that predominantly affects immunocompromised people and is relatively uncommon in immunocompetent individuals. Acute toxoplasmosis primarily presents with focal and diffuse neurological signs and symptoms depending on the site of the lesion, the degree of local damage, and the severity of inflammation. In this report, we present a case of cerebral toxoplasmosis in an immunocompetent adult female who presented with an altered level of consciousness, fever, headache, and shortness of breath. This case highlights the diagnostic challenges that may arise when dealing with patients who have a wide range of clinical manifestations.

## Introduction

*Toxoplasma gondii*, an obligate intracellular protozoan, causes toxoplasmosis, an acquired infection that is generally asymptomatic in healthy people, causing self-limited, bilateral, symmetrical, non-tender lymphadenopathy or a mononucleosis-like illness in about 10% of the population [1]. Toxoplasmosis represents 60% of localized intracerebral masses in patients with acquired immunodeficiency syndrome (AIDS) and rarely occurs in immunocompetent individuals [1]. Patients who develop deficient cell-mediated immunity, such as those with AIDS, are at risk of reactivating latent toxoplasmosis infection, which can manifest as cerebral toxoplasmosis [2]. However, this infection is extremely rare in patients without an underlying immunodeficiency. Our case report describes an immunocompetent middle-aged woman who was diagnosed with cerebral toxoplasmosis after thorough negative investigations and workup.

## Case presentation

A 30-year-old housewife, married with three children and a resident of Hyderabad, Sindh, Pakistan, known to have hypertension for the last six years, presented to the outpatient department of oral and maxillofacial surgery at a tertiary care hospital in Sindh, Pakistan with complaints of an insidious onset of altered level of consciousness (ALOC) and inability to swallow for five days, bleeding from the mouth for 10 days, and shortness of breath (SOB) with fever for two weeks. Her ALOC was gradual in onset, at first she could recognize family despite being drowsy but later proceeded to be completely obtunded. There had previously been episodes of losing consciousness in the last two months with regaining consciousness within a few minutes. Her fever was continuous, low-grade in nature, and documented around 101°F on two different occasions during that period. The patient developed SOB, which was progressive, accompanied by easy fatigability, generalized pallor, and fever. On further inquiry, the patient reported two episodes of oral bleeds that were copious in quantity, odorless, fresh, and bright red. A week after the bleeding episode, she noticed her SOB worsening and affecting her daily chores. It progressed from grade 1 to 4 over the course of a week until she presented to the hospital. The patient had been feeling tired for the last two months before the onset of symptoms, but because it was not significant enough to hinder her daily life activities, she did not seek medical attention. The patient also complained of a bilateral, diffuse headache, which was not associated with aura, photophobia, phonophobia, or ear discharge. Her vision gradually worsened, and blurriness increased bilaterally to the point where she could not tell the difference between two things. The patient was referred to the internal medicine department of our hospital for further workup and management. The family and the patient reported no history of seizures or vomiting. There was no history of vomiting, coughing, heartburn, or rashes. The patient had a history of developing gestational hypertension during her third pregnancy six years ago. Her blood pressure had been on the higher side since then but she was not taking any anti-hypertensive medications. The patient had a history of multiple blood transfusions as well. There was no history of tobacco smoking or other addictions, keeping pets, recent exposure to animal waste, or recent exposure to undercooked food that the patient or her family could recall.

On general physical examination, a middle-aged visibly distressed lady of average height and build, responding to pain and loud vocal commands, was seen with icterus, generalized rash, purpura on all her extremities, and oral ulcers. No lymphadenopathy, skin tightening, or joint swelling was noted. Blood pressure, respiratory rate, and pulse were regular and within normal limits. A temperature of 100°F was documented. The oral ulcers involved the labial folds, buccal mucosa, tongue, and posterior pharyngeal wall and appeared as punched-out lesions. A grade 2 murmur was noticed on cardiovascular examination, radiating to the entire precordium and coinciding with the carotid pulse. Neurological examination showed that both pupils were equal and reactive to light; however, there was slurred speech and an altered mental state. The examination of her cranial nerves was grossly unremarkable. No signs of meningeal irritation or neck rigidity were noticed. She had a Glasgow Coma Scale score of 8/15. Power was 3/5 in the right upper and lower extremities and 2/5 in the left upper and lower extremities, whereas hyporeflexia (+1) was noted in all extremities. The right plantar was up-going, while the left was normal (down-going). Ophthalmic examination showed scleral erythema and petechial hemorrhages bilaterally in the conjunctiva. Respiratory examination showed tachypnea with labored breathing, wheezing, and mild rhonchi in both lower lobes. However, a chest X-ray did not reveal any significant findings. However, we did not proceed with a CT scan of the chest given the financial constraints for the patient.

**Figure 1:**
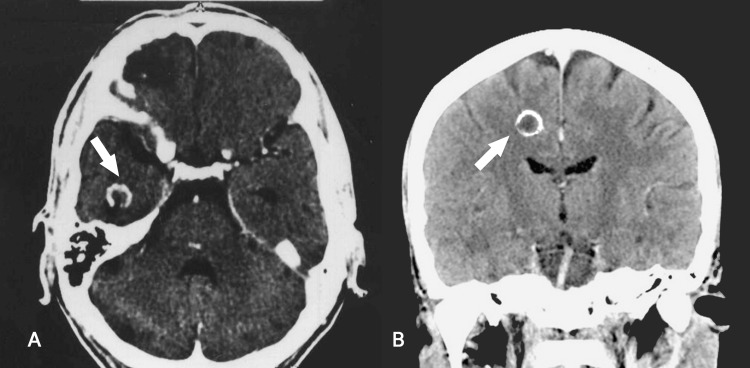
CT scan of the brain showing a ring-enhancing, intracerebral lesion (white arrow) in the (A) right temporal lobe and (B) parietal lobe.

A provisional diagnosis of lupus cerebritis was made while radiological and hematological investigations were underway for further evaluation. Her complete blood count showed anemia, thrombocytopenia, and leukocytosis. Her arterial blood gases showed an alkalotic picture. Hepatitis B surface antigen, anti-hepatitis C virus RNA, and human immunodeficiency virus (HIV) (information and communication technology) were negative, whereas her anti-hepatitis D virus was positive. Serum lactate dehydrogenase was 1,461 U/L, and her IgE level was 440.6 IU/mL. Her anti-nuclear antibody profile was negative as well. Her urinalysis showed increased red blood cells with nephritic-range proteinuria (<3 g/dL). Echocardiography was done to further assess the finding of a murmur and rule out infective endocarditis and mural or vegetative thrombi leading to sepsis and cerebral embolism. Mild mitral regurgitation and mild systolic dysfunction were noticed. Further, a CT scan of the brain revealed multiple ring-enhancing, intracerebral, space-occupying lesions in the right parietal, temporal, and frontal lobes (Figure 1).

Anti-Toxoplasma IgG antibodies were found to be elevated up to 48.5 IU/mL (normal = <6.5 IU/mL), which helped in confirming cerebral toxoplasmosis as the final diagnosis. The patient was started on co-trimoxazole 10 mg/kg intravenously for three weeks followed by an oral dose of co-trimoxazole of 960 mg daily for three weeks more. The patient was symptom-free after six weeks; however, lesions seen in her MRI were healing gradually when the patient was called for follow-up after three months.

## Discussion

The reactivation of latent infection frequently causes cerebral toxoplasmosis with necrotizing encephalitis within the brain in immunocompromised individuals. It is the most prevalent opportunistic illness in AIDS patients in both developed and developing nations [3]. The causative agent, *Toxoplasma gondii*, can be transmitted in humans either by ingestion of cysts or oocysts released in feline feces, ingestion of contaminated or inadequately cooked meat, or via vertical transmission [4]. When mammals ingest the sporozoites released by oocyst, they develop into tachyzoites which can disseminate into the blood and infect nucleated cells in humans, primarily lymph nodes, central nervous system, retinal cells, lungs, and heart [5]. The absence of feline contact or a history of ingestion of uncooked food in our patient made diagnosing the disease particularly challenging.

In immunocompromised patients, toxoplasmosis is a life-threatening condition and produces a wide range of clinical manifestations. On the other hand, only approximately 10% to 20% of immunocompetent patients become symptomatic. Previous cases of cerebral toxoplasmosis have been reported in non-immunocompromised pregnant females, patients undertaking immunosuppressive therapy for their chronic diseases, and, rarely, in immunocompetent patients [6,7]. Our patient, an immunocompetent female, tested negative for pregnancy and HIV and had no history of immunosuppressive treatment. Hoti et al. [8] reported a similar case of intracranial toxoplasmosis in an immunocompetent patient presenting with signs of increased intracranial pressure but no prior immunosuppressive history.

An immunocompetent patient with toxoplasmosis may show signs and symptoms of lymphadenopathy, fever, malaise, and myalgias, whereas immunocompromised patients can have neurological symptoms such as headache, seizures, focal neurologic deficits, cranial nerve deficits, disorientation, encephalitis, and meningoencephalitis. Our patient presented with ALOC, impaired vision, speech, and headache [4]. Acute toxoplasmosis can also cause pulmonary symptoms in immunocompromised patients, but such an occurrence is rare in immunocompetent patients, with only a few cases documented in the medical literature. There have also been reports of myocarditis in toxoplasmosis patients [4]. There are no known predisposing factors for toxoplasmosis pneumonia; however, infections with more virulent strains, a large parasite load, or even microbe inhalation are possible explanations [5].

Diagnostic challenges are very common in such complex clinical presentations, specifically in immunocompetent patients. Our patient also met a diagnostic dilemma and various diagnoses including disseminated tuberculosis, HIV encephalopathy, aspergillosis, progressive multifocal leukoencephalopathy, bacterial abscess, cryptococcosis, and infectious endocarditis with cerebral emboli were provided before a final diagnosis could be made considering the patient’s age, immunological status, sub-acute clinical deterioration, a negative oncology history, and a thorough blood workup.

Contrast-enhanced CT or MRI scans often reveal multiple nodular or ring-enhancing lesions associated with cerebral toxoplasmosis. These lesions are frequently accompanied by vasogenic edema that is disproportionate to their size, causing a mass effect. The basal ganglia and frontal and parietal lobes are the most common locations for these lesions, but they can also affect other areas, such as the brain stem/cerebellum, temporal lobe, and occipital lobe. In our case, multiple ring-enhancing lesions were identified in the frontal, parietal, and temporal lobes as well but the patient was not immunocompromised [9,10]. Serological measurement of IgM and IgG antibody levels is the most used approach for diagnosing this infection [11], which was also the prime method of testing in our patient apart from the definitive cerebrospinal fluid biopsy. Pyrimethamine-sulfadiazine and co-trimoxazole both have been demonstrated to be effective in immunocompetent individuals [12]. Our patient was treated with co-trimoxazole for six weeks and remains on outpatient follow-up.

## Conclusions

Cerebral toxoplasmosis is a life-threatening condition that primarily affects immunocompromised patients and is rarely seen in immunocompetent individuals. Cerebral toxoplasmosis should be considered an important differential in patients with neurological deficits and space-occupying lesion(s) on CT or MRI without any other known cause. Contrary to our case, this infection usually follows a benign course in immunocompetent individuals. Early detection and appropriate medical management are crucial to prevent further clinical deterioration and the development of complications.
